# Comprehensive Radiological Imaging for the Characterization of Spinal Dysraphism and Associated Anomalies in a Pediatric Case

**DOI:** 10.7759/cureus.68415

**Published:** 2024-09-01

**Authors:** Iffath Misbah, Pranathi Ravula, Sam Raja, Arunkumar Mohanakrishnan, Paarthipan Natarajan, Dhivya Gunasekaran

**Affiliations:** 1 Radiodiagnosis, Saveetha Medical College and Hospital, Saveetha Institute of Medical and Technical Sciences (SIMATS), Chennai, IND

**Keywords:** tethered spinal cord, butterfly vertebrae, sprengel’s deformity, scoliosis, type 2 diastematomyelia, spinal dysraphism

## Abstract

Spinal dysraphism is a spectrum of congenital anomalies caused by incomplete neural tube closure during early development, leading to spine and spinal cord defects. These can be broadly categorized into anomalies of gastrulation (including disorders of notochord formation and integration), anomalies of primary neurulation (such as premature disjunction and nondisjunction), combined anomalies of gastrulation and primary neurulation, and anomalies of secondary neurulation. This case report focuses on a 15-year-old male patient who exhibits a range of congenital spinal anomalies of spinal dysraphism spectrum, each contributing to a complex clinical picture. The primary aim of this report is to highlight the critical role of multimodal imaging in the evaluation of such conditions. Detailed imaging studies, particularly magnetic resonance imaging (MRI), are indispensable in accurately diagnosing, guiding surgical planning, and managing the diverse anomalies associated with spinal dysraphism. In this case, imaging findings were pivotal in identifying multiple congenital abnormalities, including scoliosis, butterfly vertebrae, block vertebrae, spina bifida occulta, and diastematomyelia. These conditions pose significant diagnostic and management challenges due to their varied presentations and complications.

## Introduction

Congenital malformations of the spine and spinal cord, known collectively as spinal dysraphism, encompass a diverse array of anomalies resulting from incomplete midline closure of osseous, mesenchymal, and nervous tissues. The spinal cord develops during early embryogenesis (between two and six weeks of gestation) through three primary stages: gastrulation, primary neurulation, and secondary neurulation. Defective primary neurulation can lead to conditions such as open spinal dysraphism (OSD), closed/occult spinal dysraphism (CSD), and dorsal dermal sinus [1-3].

In contrast, defects during secondary neurulation are associated with filar lipoma, tight filum terminale, caudal agenesis, and sacrococcygeal teratoma [4,5]. Additionally, improper development of the notochord can result in diastematomyelia, neurenteric cyst, caudal agenesis, and segmental spinal dysgenesis [6-8]. Caudal or sacral agenesis, which involves the malformation of the distal spinal segments, can result in the partial or total absence of sacral vertebrae, profoundly affecting mobility and overall quality of life [9,10]. Features like colpocephaly can indicate corpus callosal agenesis, while interdigitating gyri suggest a fenestrated falx.

Block vertebrae and butterfly vertebrae are vertebral anomalies linked to spinal dysraphism. Block vertebrae are defined by the congenital fusion of adjacent vertebrae, while butterfly vertebrae occur when the vertebral body is formed by the fusion of two lateral sclerotomes from the somites. The failure of these sclerotomes to fuse properly leads to the development of butterfly vertebrae [11-13].

Scoliosis is characterized by one or more lateral curves of the vertebral column in the coronal plane, though abnormal curves can impact spinal alignment in all three dimensions. Scoliosis often accompanies spinal dysraphism and presents notable clinical challenges [14,15].

These anomalies can occur independently, and the simultaneous presence of all these conditions is extremely rare. We present the case of a 15-year-old male patient who had not undergone any medical investigations since birth. Upon conducting radiological examinations, we unexpectedly discovered the presence of multiple types of spinal dysraphism coexisting in this patient.

## Case presentation

Here we present a case of a 15-year-old male who initially presented with a high-grade fever lasting one week, which responded well to medication. On thorough general and systemic examination, the child was noted to be malnourished and moderately built, with a height of 143 cm (below the second percentile) and a weight of 23 kg (below the third percentile). He was the first-born male child, born preterm at 36 weeks of gestation, with a history of a five-day neonatal intensive care unit (NICU) admission after birth. His intellectual functioning was notably below average for his age group.

The clinical examination highlighted a pronounced abnormal lateral curvature of the spine, presenting an "S" shape, indicative of scoliosis. There was a significant asymmetry between the scapulae, particularly a high-riding left scapula, which was visually prominent. Other notable physical findings included impaired muscle control on the left side of the body, an uneven waistline, and a tuft of hair located in the lumbar region, suggestive of possible underlying spinal abnormalities.

There is no documented family history of similar conditions, which makes this case particularly unique. Despite these challenges, the patient currently manages daily activities, attends school, and is actively involved in ongoing orthopedic and neurosurgical consultations aimed at addressing his complex congenital anomalies. These consultations are crucial for developing a comprehensive management plan tailored to his specific needs and ensuring the best possible outcomes for his condition.

Imaging features

**Figure 1:**
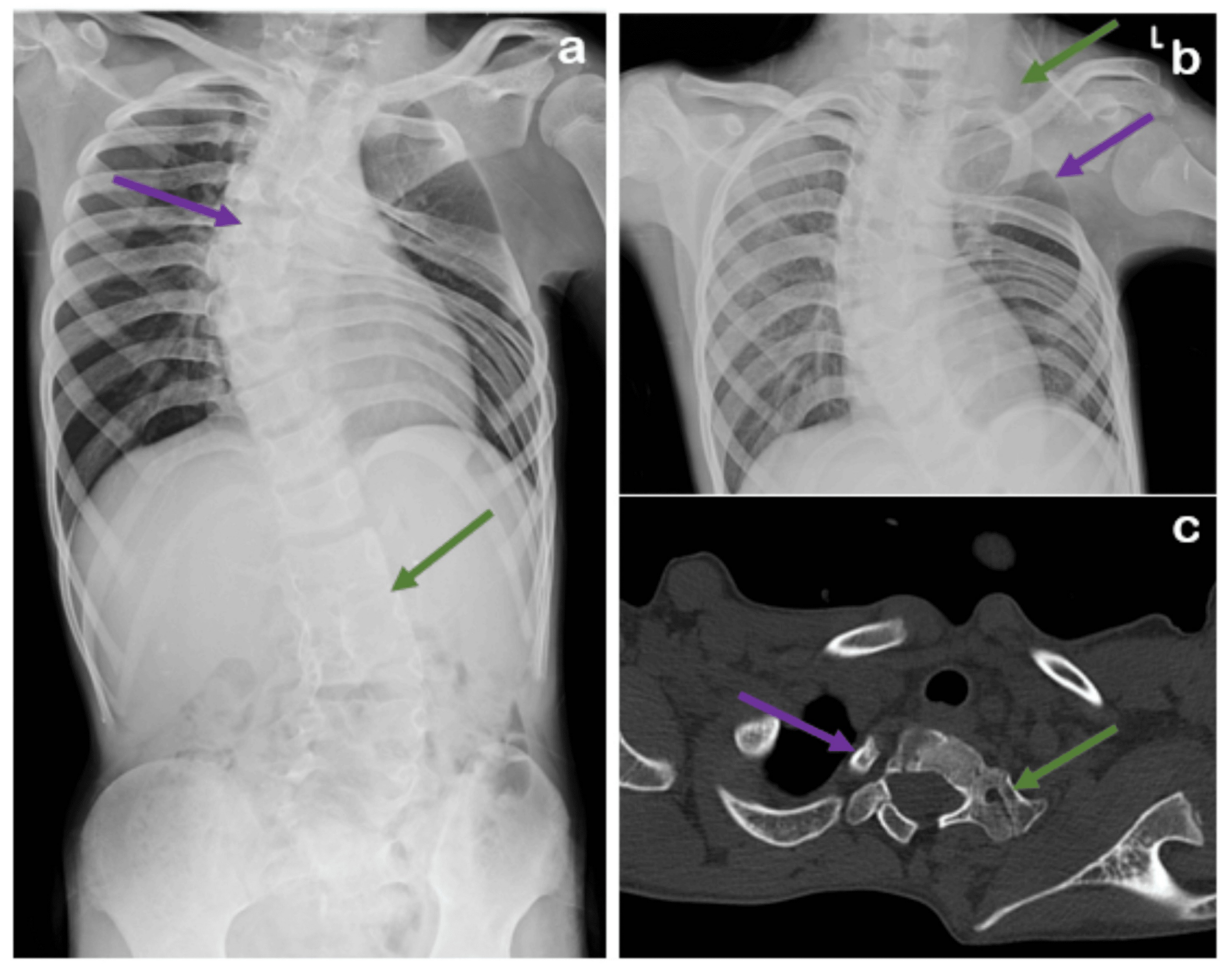
(a) Radiograph shows dextroscoliosis of the dorsal spine (violet arrow) and levoscoliosis of the lumbar spine (green arrow). (b) Chest radiograph reveals a high-riding left scapula (green arrow) and aplasia of the left second rib (violet arrow). (c) CT axial image illustrates bilateral cervical ribs (violet and green arrow).

**Figure 2:**
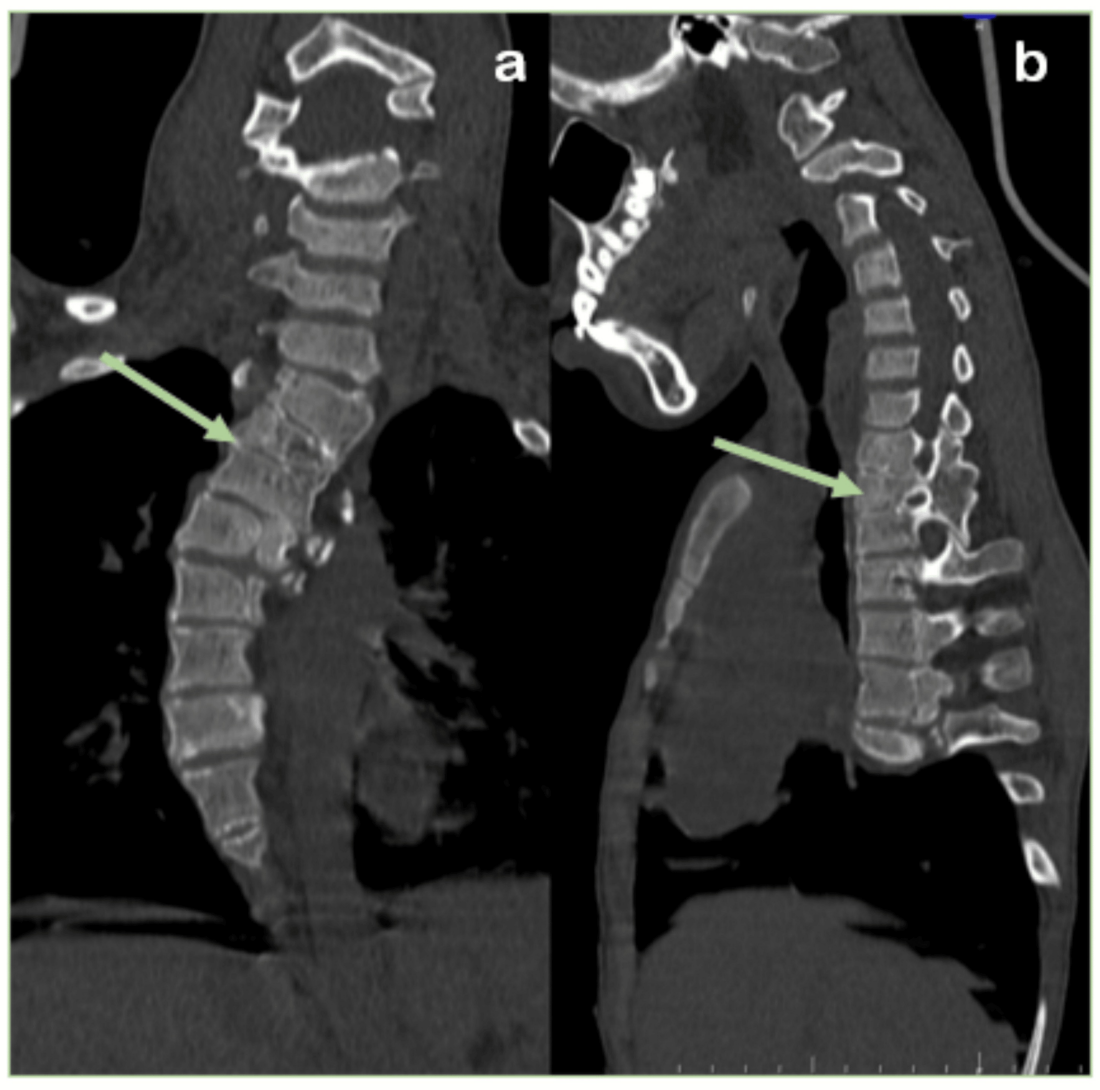
Computed tomography: (a) spine coronal and (b) sagittal images show congenital dorsal block vertebrae (green arrows).

**Figure 3:**
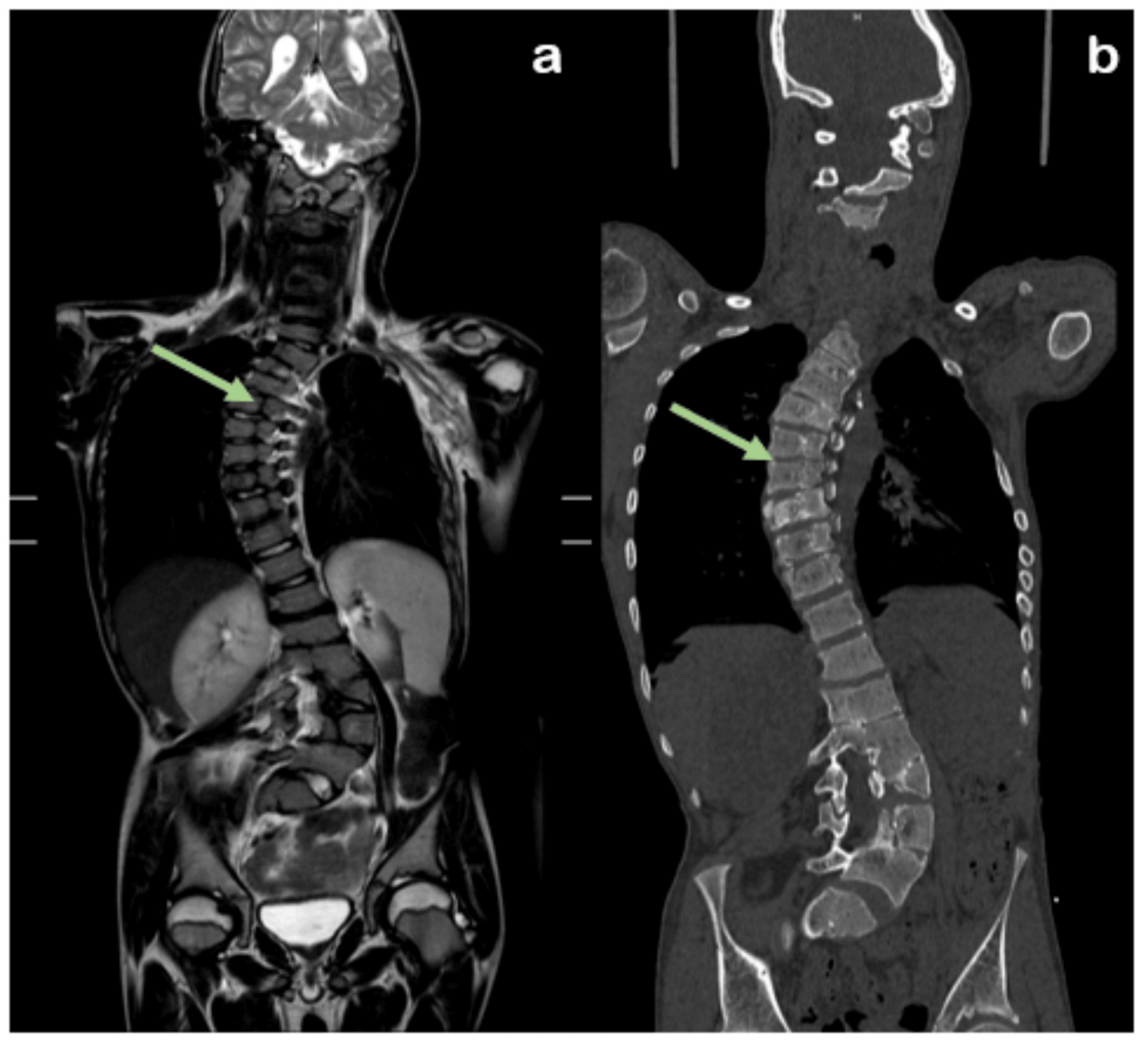
(a) Coronal section images of the spine from magnetic resonance imaging (T2 sequence) and (b) computed tomography scans reveal butterfly vertebrae (green arrow) extending from the D4 to L2 vertebral levels.

The patient was subjected to radiological investigations where chest radiography (CR) revealed dextroscoliosis of the dorsal spine from D2 to D9 vertebral levels with convexity toward the right and levoscoliosis of the lumbar vertebrae from D12 to L5 with convexity toward the left. The left scapula was elevated and rotated, with the inferior angle directed laterally, likely Sprengel's deformity (no omovertebral bar). Computed tomography (CT) showed bilateral cervical ribs with aplasia of the left second rib and congenital partial block vertebra was noted from D1-D2 to D3-D4, L1-L2, and L5-S1 vertebral levels. Magnetic resonance imaging (MRI) and CT illustrating dorsolumbar butterfly vertebra was noted from D4 to L2 vertebral levels (Figures 1-3).

Findings from CT and MRI scans

**Figure 4:**
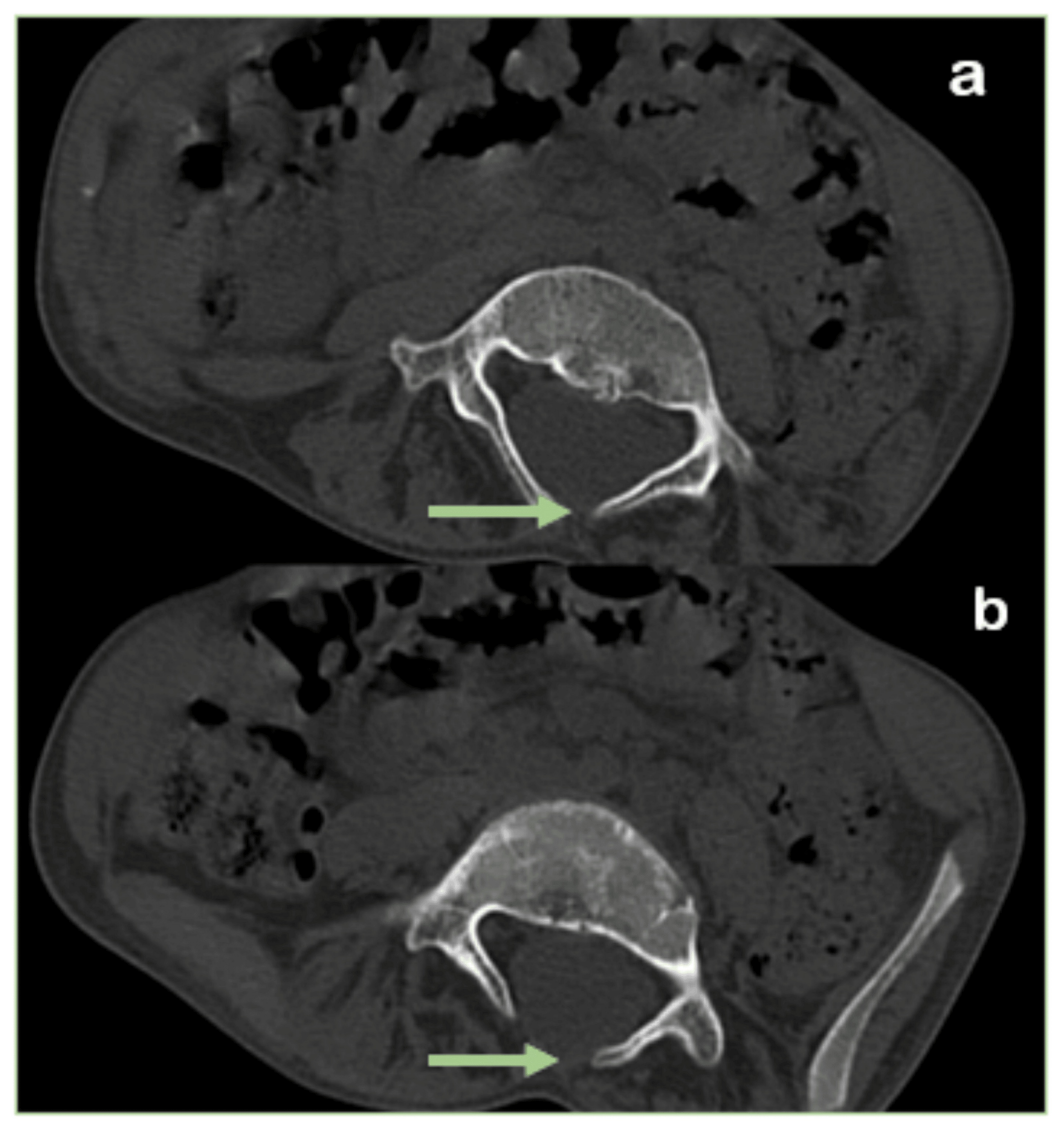
Computed tomography axial section images (a,b) of the spine reveal an unfused spinous process of lumbar vertebrae (green arrow).

**Figure 5:**
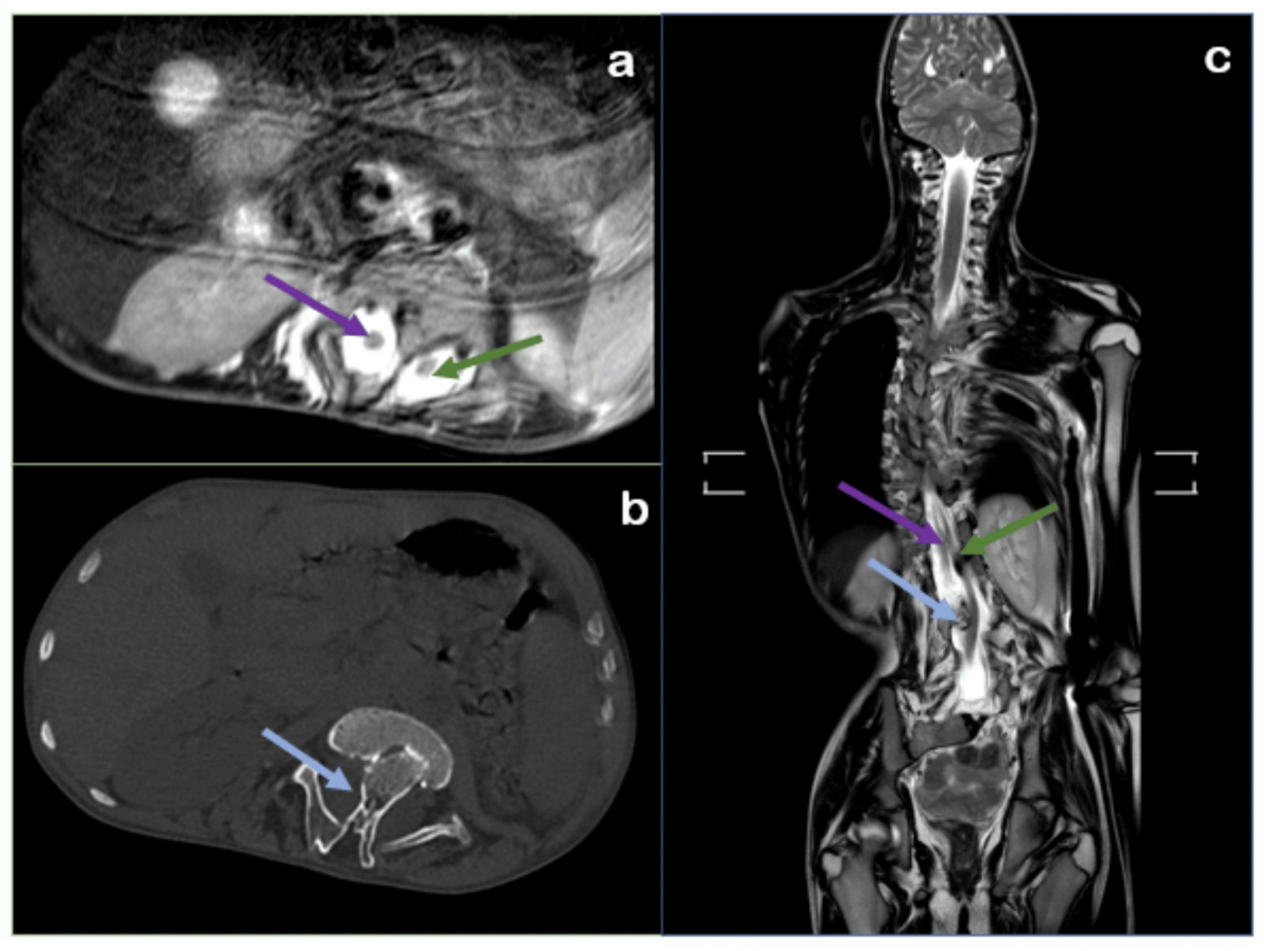
Axial and coronal section images of the spine from MRI T2 sequence: (a, c) show the presence of hemicord-1 (violet arrows) and hemicord-2 (green arrows), while CT axial (b) and MRI coronal images (c) illustrate a bony septum (blue arrows) between the hemicords.

CT images of the spine show a defect in the posterior elements of the lumbosacral vertebrae (L2-L4), involving the spinous process, suggestive of spina bifida occulta. MRI demonstrates the presence of two hemicords, while CT images reveal a bony septum between them. Additionally, the MRI image shows a duplicated dural sac extending from D11-D12 to L2-L3, with two distinct hemicords and an osseous septum at the L1-L2 level, which is characteristic of type I diastematomyelia (Figures 4, 5).

**Figure 6:**
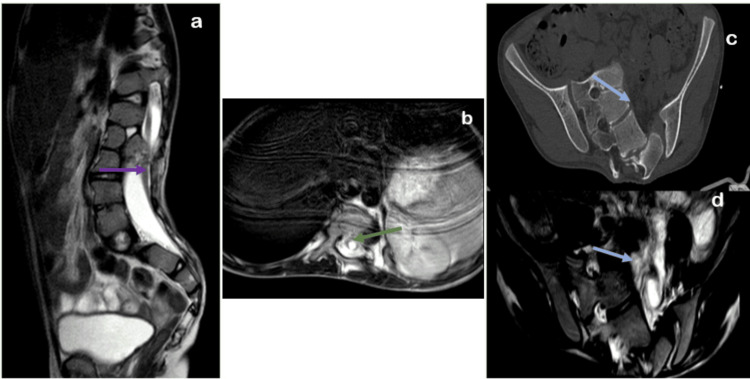
MRI spine T2 sequence: (a) sagittal image shows a low-lying tethered cord (violet arrow), (b) axial image shows syrinx/hydromyelia (green arrow). Axial section images from CT (c) and MRI T2 sequence (d) reveal partial unilateral left sacral agenesis (blue arrow).

**Figure 7:**
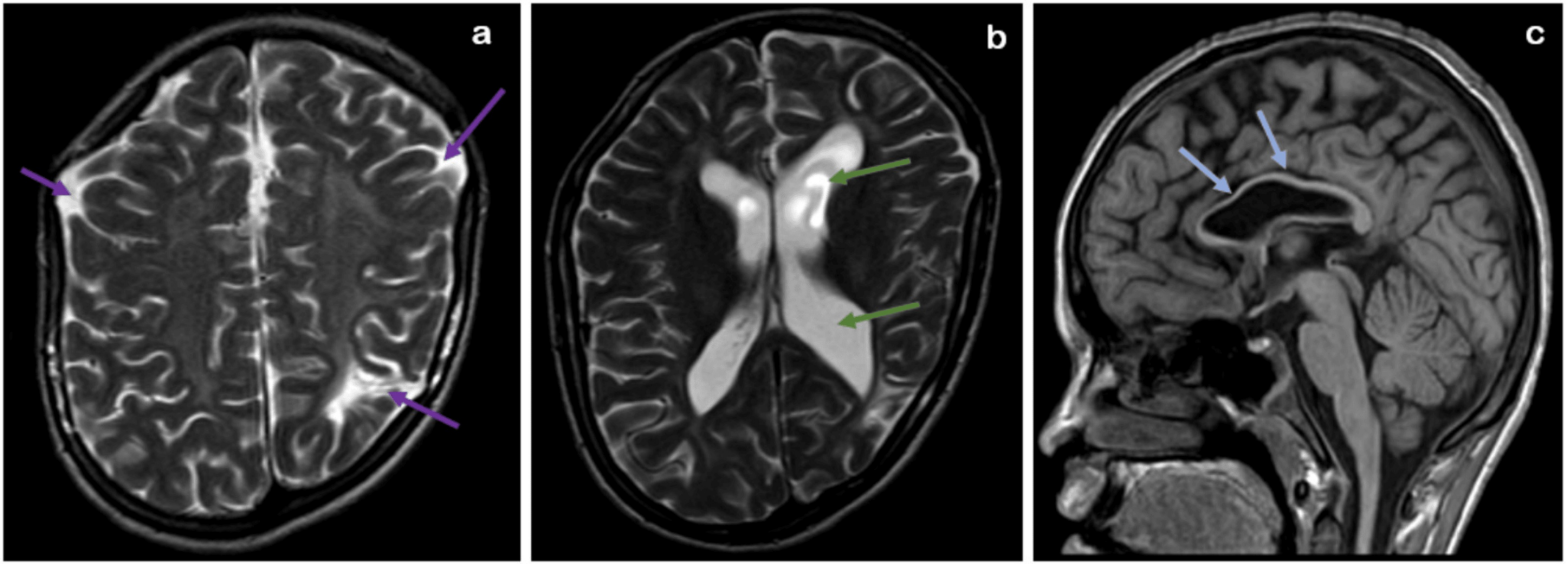
MRI brain T2 sequence axial images (a,b) demonstrate gliosis with encephalomalacic changes in the bilateral frontal and left parietal lobes (violet arrows), dilated left lateral ventricle (green arrows) and (c) T1 sequence coronal image shows a thinned genu and body of the corpus callosum (blue arrows).

MRI images of the spine reveal a low-lying tethered cord and a syrinx/hydromyelia. The CT and MRI scans show a partial unilateral left sacral agenesis at the S1, S2, and S3 vertebral levels. The MRI brain images demonstrate gliosis with encephalomalacic changes in the bilateral frontal and left parietal lobes, a dilated left lateral ventricle, and a thinned genu and body of the corpus callosum (Figures 6, 7).

## Discussion

Spinal dysraphism, a complex and diverse group of congenital anomalies, arises from incomplete neural tube closure during embryogenesis. These anomalies, including spina bifida occulta (SBO) and various forms of closed spinal dysraphism (CSD), present significant challenges in diagnosis and management [1,2]. SBO is characterized by a defect in the vertebral laminae that does not affect the spinal cord or meninges, marked by intact skin and external markers such as hair tufts, hemangiomas, and pigmented spots, includes conditions like split cord malformations, thickened filum terminale, spinal lipomas, dermoid cysts, and syringohydromyelia [3,4]. The fusion of paired notochordal anlagen into a single midline structure, known as midline notochordal integration, is crucial for normal spinal development. Abnormalities during this stage can result in longitudinal splitting of the spinal cord, leading to conditions such as diastematomyelia [5,6]. Diastematomyelia, or split cord malformation, involves the division of the spinal cord into two hemicords, which can be either symmetric or asymmetric, each contained within its own dural sac. This condition is often complicated by a midline osseous or osseocartilaginous spur, resulting in scoliosis, tethered cord syndrome, and vertebral anomalies such as hemivertebrae and butterfly vertebrae [7,8].

Detailed imaging capabilities of MRI are essential for identifying the duplicated dural sac and the nature of the bony spur, which are critical for surgical planning and management. A thickened and shortened filum terminale causes tethering of the spinal cord, hindering the ascent of the conus medullaris. This defect, arising from abnormal retrogressive differentiation during secondary neurulation, is identified on imaging by a thickened filum terminale (greater than 2 mm) and a low-lying conus medullaris below the L2 vertebral body [9]. This condition is often associated with other malformations, and isolated cases are rare. Caudal or sacral agenesis is another significant anomaly within the spectrum of spinal dysraphism. This rare condition impacts the formation of the distal spinal segments, potentially resulting in partial or complete absence of sacral vertebrae [10]. Additional vertebral anomalies, such as block vertebrae and butterfly vertebrae, further complicate the clinical picture. Block vertebrae involve the congenital fusion of adjacent vertebrae, while butterfly vertebrae occur when the vertebral body forms from the fusion of two lateral sclerotomes derived from somites. The failure of these sclerotomes to fuse results in a sagittal cleft within the vertebral body, contributing to spinal deformities and instability [11-13].

Scoliosis, defined by one or more lateral curves of the vertebral column in the coronal plane, frequently accompanies spinal dysraphism and presents significant clinical challenges [14]. Both dextroscoliosis and levoscoliosis can exacerbate the patient's condition, requiring a comprehensive approach to management. The detailed imaging provided by MRI is indispensable in diagnosing the extent and nature of these anomalies and planning effective treatment strategies [15]. Sprengel's deformity, characterized by a high-positioned scapula due to failed caudal migration during embryogenesis, often coexists with spinal dysraphism. This high scapular position results from a failure of normal descent during embryonic development, influenced by muscular actions that may be insufficient due to excessive intrauterine pressure or defective musculature. The scapulae appear around the fifth week of embryogenesis at the level of the fifth cervical and first dorsal vertebrae and gradually descend to their normal position between the second and seventh dorsal vertebrae. The use of MRI in diagnosing and managing spinal dysraphism and its associated anomalies is crucial [16,17].

MRI provides comprehensive and detailed images necessary for accurate diagnosis, effective management, and optimal patient outcomes. This case report emphasizes the importance of a thorough, multimodal diagnostic approach in managing complex spinal dysraphism cases, highlighting MRI's pivotal role in modern clinical practice. The most common surgery for diastematomyelia is decompression surgery, which involves removing the thin piece of bone or cartilage that separates the spinal cord [18,19]. This procedure creates more space for the spinal cord within the spinal column. In many instances, this removal also "un-tethers" the spinal cord, enabling it to move freely again [20]. Growth-modulated constructs, including growing rods, vertical expandable prosthetic titanium ribs, and vertebral body tethering, can be utilized to dynamically correct scoliosis deformities as the patient grows [21]. 

## Conclusions

This case report highlights the importance of a comprehensive, multimodal diagnostic approach, emphasizing the pivotal role of MRI in modern clinical practice to achieve optimal patient outcomes. Effective management of spinal dysraphism necessitates a coordinated effort among radiologists, orthopedic surgeons, and neurosurgeons to address the multifaceted challenges posed by these congenital anomalies. Through a detailed imaging and clinical evaluation, early diagnosis and tailored interventions can be implemented, potentially improving the quality of life of the affected individuals. The integration of advanced imaging techniques, such as CT and MRI, facilitates precise anatomical and functional assessments, guiding surgical planning and postoperative care. Continuous collaboration and interdisciplinary communication remain essential in advancing the understanding and treatment of spinal dysraphism.
